# The role of exosomal microRNAs; focus on clinical applications in breast cancer

**DOI:** 10.20517/cdr.2019.17

**Published:** 2019-09-19

**Authors:** Aiko Sueta, Yutaka Yamamoto, Hirotaka Iwase

**Affiliations:** Department of Breast and Endocrine Surgery, Kumamoto University Graduate School of Medical Sciences, 1-1-1, Honjo, Chuo-ku, Kumamoto 860-8556, Japan.

**Keywords:** Exosomal miRNA, breast cancer, liquid biopsy

## Abstract

Despite several advances in targeted therapies for breast cancer, breast-cancer-associated death remains high in women. This is partially due to the lack of reliable markers predicting metastatic disease or recurrence after initial therapy. Recent research into the clinical validity of circulating cancer-specific biomarkers as a “liquid biopsy” is of growing interest. Of these, exosomal microRNAs (miRNAs) are promising candidate biomarkers for clinical use in breast cancer. In addition to their diagnostic value, exosomal miRNAs play an important role in predicting clinical outcome or treatment response. In this review, it is focused on the findings concerning exosomal miRNAs in relation to disease detection, prognostic impact and therapeutic effect in breast cancer, and discuss their clinical utility.

## Introduction

Breast cancer is one of the most commonly-diagnosed cancers worldwide and one of the leading causes of cancer death in women^[1]^. Improvements in the early detection of breast cancer by conventional screening programs or development of novel molecular-targeted therapy may contribute to the reduction of breast cancer-related death. Breast cancer represents a heterogenous disease with varied biological characteristics and clinical outcomes^[2]^. Based on molecular subtypes, namely, the expression of estrogen receptor (ER), progesterone receptor and human epidermal growth factor receptor-2 (HER2), the disease is classified into four distinct subtypes; luminal A, luminal B, HER2-enriched, and triple negative (TN)^[3,4]^. A better understanding of these subtypes is required to determine the need for appropriate therapy, such as endocrine therapies, chemotherapy, and molecular-targeted therapies. Although these therapies are commonly used for early breast cancer patients, some have experienced resistance following therapy, leading to loco-regional or distant recurrence^[5,6]^. Many efforts have been made to explore the potential of various biomarkers to predict clinical outcome or treatment response^[7-9]^, nevertheless, the mechanisms underlying the acquisition of resistance to therapy are still poorly elucidated.

Currently, clinical determinations of treatment are based on biopsies of tumor tissues, that may not be representative of the entire tumor burden^[10,11]^. To overcome the problem of tumor heterogeneity, the novel diagnostic tool which is referred to as a “liquid biopsy” is an ideal methodology^[12,13]^. In recent years, there have been a number of advances in the field of molecular profiling to accurately predict clinical outcome and treatment response in early breast cancer^[14]^. Circulating cancer-specific biomarkers can be evaluated by minimally-invasive techniques and are easily accessible to analyze compared to tissue sampling, as well as providing a real-time assessment^[15]^. To date, several candidate biomarkers, including RNA, microRNA (miRNA), DNA, and proteins, have been suggested^[16]^.

MiRNAs are single-stranded and non-coding RNAs with 19 to 22 nucleotides in length, which regulate gene expression at the post-transcriptional level by binding to partially-complementary sites in the 3’-untranslated regions of target messenger RNAs (mRNAs), leading to translational repression or regulation of mRNA degradation^[17,18]^. Bioinformatic techniques have revealed that each miRNA has binding affinity to hundreds of target genes^[19]^. They are known to act functionally as oncogenes or tumor suppressor genes. Besides their intracellular function, there are some evidences of miRNA activity in cell to cell communication^[20]^. In breast cancer, the expression of miRNAs is frequently deregulated during tumorigenesis as well as various types of cancer^[21]^. To date, numerous miRNAs are reported to be valuable biomarkers for critical regulators of tumor initiation, metastasis and chemoresistance^[22-24]^. In particular, circulating miRNAs can be found in several different body fluids and reflect specific disease types. After released into the blood circulation, miRNAs are commonly incorporated into microvesicles, bound to lipoproteins such as HDL, or form complexes with Argonaute-2 (Ago2) proteins^[25]^. It has been suggested that the majority of miRNAs in circulation are selectively concentrated in exosomes^[26,27]^ and exosomal miRNAs are considered the main source of circulating miRNAs.

Exosomes are small membranous vesicles with a diameter of 30-100 nm and consisting of lipid, proteins, DNA, miRNAs and mRNAs. They are actively secreted from various types of cells into the circulation^[20]^. They can be transferred from primary tumor cells to distant organs via the circulation; thus, they reflect the origin of the secreting cells. The cancer-derived exosomes may be important mediators of intercellular communication^[28,29]^ and the presence of miRNA in exosomes was first reported by Valadi *et al*.^[20]^. Exosomal miRNAs are the most abundant and crucial biomolecules which play a pivotal role in implementing the tumor microenvironment and the growth and metastasis of cancer cells. They have been proposed as novel circulating biomarkers for the early detection of several types of cancers^[30-32]^, based on findings indicating that cancer patients have greater amounts of circulating exosomes than those in healthy controls^[33,34]^.

There have been many reports on circulating miRNAs, whereas limited data are available regarding exosomal miRNAs. In this review, we focus on the role of exosomal miRNAs as markers for possible applications in breast cancer detection, prognosis, and response to treatment. Moreover, we discuss the issue of exosome research in terms of clinical application.

## Diagnostic potentials of exosomal micrornas

Exosomal miRNAs in body fluids have potentials of the novel circulating biomarkers for the detection of several cancers^[30-32]^, based on findings indicating that cancer patients have elevated levels of tumor-derived exosomes in blood, compared with those in healthy controls^[33,34]^. To date, there have been few studies involving analysis of exosomal miRNAs in the diagnosis of breast cancer [Table 1]. In one study, miR-1246 and miR-21 in plasma exosomes were reported to be useful indicators of breast cancer diagnosis^[35]^. These miRNAs could successfully differentiate breast cancer patients from healthy control subjects. The area under the curve (AUC) was 0.73 to differentiate them when miR-1246 and miR-21 were combined in the analysis, that is considered moderate performance. MiR-1246 suppressed the expression of Cyclin G2, leading to breast cancer progression. A recent study by Li *et al*.^[36]^ indicated the potential use of an exosomal miR-106a-363 cluster as a novel diagnostic biomarker. Four plasma-derived exosomal miRNAs (miR-106a-3p, miR-106a-5p, miR-20b-5p, and miR-92a-2-5p), and four serum-derived miRNAs (miR-106a-5p, miR-19b-3p, miR-20b-5p, and miR-92a-3p) from breast cancer patients were expressed at significantly higher levels than those from healthy controls. Interestingly, two overlapping miRNAs (miR-106a-5p and miR-20b-5p) were consistently upregulated also in breast cancer tissues. The functional mechanisms of the miR-106a-363 cluster in breast cancer have not been fully reported, whereas all but one miRNA (miR-106a-3p) are involved in the “proteoglycans in cancer” pathway according to the Kyoto Encyclopedia of Genes and Genomes (KEGG) pathway analysis.

**Table1:** Exosomal miRNAs as diagnostic markers for breast cancer

miRNAs	Sample increased expressions	Indicator	Ref.
miR-1246, miR-21	Patient plasma sample Plasma of PDX	Increased expressions are biomarkers of detection for breast cancer	[35]
miR-106a-3p, miR-106a-5p, miR-20b-5p, and miR-92a-2-5p	Patient plasma sample	Increased expressions are biomarkers of detection for breast cancer	[36]
miR-106a-5p, miR-19b-3p, miR-20b-5p, and miR-92a-3p	Patient serum sample	Increased expressions are biomarkers of detection for breast cancer	[36]
miR-101, miR-372	Patient serum sample	Increased expressions are biomarkers of detection for breast cancer	[27]
miR-373	Patient serum sample	Exosomal miR-373 is higher in receptor-negative and TN tumors than in hormone receptor-positive cancer	[27]
miR-335, miR-628, and miR-422a	Patient plasma sample	Combined scores could discriminate between triple negative- and HER2-positive breast cancer patients	[37]
miR-223-3p	Patient plasma sample	The expression level is higher in the patients with IDC than DCIS	[38]
miR-16, miR-30b, and miR-93	Patient plasma sample	MiR-93 was higher in DCIS patients than in healthy women, but lower in IDC patients	[39]

PDX: patient-derived orthotopic xenograft models; IDC: invasive ductal carcinoma; DCIS: ductal carcinoma *in situ*

Differential expressions of subtype-specific miRNAs have also been reported in several studies^[27,37]^. Eichelser *et al*.^[27]^ analyzed circulating cell-free and exosomal miR-101, miR-372, and miR-373 in preoperative blood serum. In analyses of 50 breast cancer patients and 12 healthy women, the levels of exosomal miR-101 and miR-372 were significantly higher in the serum of patients with breast cancer than in healthy controls. Moreover, the levels of circulating exosomal miR-373 were higher in receptor-negative and TN tumors than in hormone receptor-positive carcinomas or healthy controls. In addition, Stevic *et al*.^[37]^ also demonstrated different exosomal miRNA signatures in HER2-positive and TN breast cancer patients. The expression levels of exosomal miRNA in plasma of 435 breast cancer patients, consisting of 211 HER2-positive and 224 TN patients, were analyzed. The analysis revealed that the expression of five miRNAs were higher and 13 were lower in HER2-positive than in TN breast cancer patients. Among them, exosomal miR-335, miR-628, and miR-422a were combined into a logistic regression model and these combined scores could discriminate between TN and HER2-positive breast cancer patients with a sensitivity 68% and a specificity of 81%. This is the first study to measure the exosomal miRNAs derived from a large cohort of 435 breast cancer patients, further indicating the different exosomal miRNA patterns among subtypes.

Several studies have compared exosomal miRNAs derived from invasive ductal carcinoma (IDC) and ductal carcinoma *in situ* (DCIS)^[38,39]^. It has been demonstrated that the *miR-223-3p* levels of IDC patients showed the highest fold-change compared with those in DCIS patients and healthy controls^[38]^. *In vitro* analysis revealed that transfection of MCF-7 cells with the *miR-223-3p* gene significantly promoted cell proliferation and cell invasion ability. In another study, exosomal miR-16, miR-30b, and miR-93 were analyzed in 111 IDC patients, 42 DCIS patients, and 39 healthy women^[39]^. The level of exosomal miR-16 was higher in the plasma of breast cancer and DCIS patients than in healthy women. Meanwhile exosomal miR-93 was higher in DCIS patients than in healthy women, but lower in IDC patients. These findings suggest that the different signatures of the corresponding miRNAs are associated with a particular biology of breast tumors. This may be promising for the identification of biomarkers if specific miRNAs could be used to distinguish DCIS cases that have a higher likelihood of future disease progression. Taken together, these findings suggest that several exosomal miRNAs could be good candidates for early breast cancer detection and differentiation of tumor subtypes.

## The role of exosomal micrornas in breast cancer development

### Exosomal miRNAs as prognostic potentials

Several miRNAs derived from exosomes can be involved in tumor metastasis and recurrence of breast cancer. There are a few articles revealing an association between exosomal miRNAs derived from breast cancer patients and clinical outcome. One of them, by Ni *et al*.^[39]^, described analysis using a PCR-based microarray which contained 47 different miRNAs in exosomes derived from the plasma of 32 breast cancer patients. They observed differences in the levels of six miRNAs between primary and recurrent breast cancer. In particular, the levels of miR-20a and miR-30b were lower in exosomes from recurrent cases than in those from primary breast cancer patients. The functions of the corresponding miRNAs are unclear, but miR-30b may regulate the Cyclin E2 gene (*CCNE2*)^[40,41]^. Ichikawa *et al*.^[41]^ demonstrated that trastuzumab induced the expression of miR-30b in breast cancer cells and upregulation of this gene inhibited cell growth by targeting *CCNE2*. According to the finding, downregulation of miR-30b may be involved in trastuzumab resistance in HER2-positive breast cancer patients. In another study, we have previously demonstrated differential expression of exosomal miRNAs between breast cancer patients with and without recurrence. Of 384 miRNAs, three (miR-338, miR-340, and miR-124) were significantly upregulated and eight (miR-29b, miR-20b, miR-17, miR-130a, miR-18a, miR-195, miR-486, and miR-93) were significantly downregulated in patients with recurrence^[42]^. In contrast to growing evidence of diagnostic potential for exosomal miRNAs, there is limited data concerning their prognostic potentials.

### Exosomal miRNAs in tumor progression

Functional studies in cell lines or xenograft models have also revealed that exosome-mediated transfer of several miRNAs has been implicated in promoting tumor growth, cell invasion and preparation of a metastatic niche [Table 2]. Accumulating evidence has indicated that recipient cells that have basically no metastatic potential could gain metastatic characteristics by receiving several miRNAs via exosomes. The miR-200 family, which regulates the mesenchymal-to-epithelial transition (MET) as well as epithelial-to-mesenchymal transition (EMT), is enriched in the circulation of patients with metastatic cancers. Le *et al*.^[43]^ revealed that taking miR-200-rich exosomes from metastatic 4T1 cells and co-culturing them with poorly-metastatic 4T07 cells resulted in transfer of miR-200 and downregulation of ZEB2 which caused the 4T07 cells to revert to an E-cadherin-expressing epithelial phenotype. Moreover, when the 4T07 cells were treated systemically with 4T1 exosomes, the incidence of lung metastasis increased. More recently, a study by Kia *et al*.^[44]^ showed that poorly-metastatic breast cancer cells (MCF-7) treated with exosomes derived from highly-metastatic breast cancer cells (MDA-MB-231) displayed overexpression of miR-9 and miR-155, which target the tumor suppressor genes, *PTEN* and *DUSP14*. They demonstrated that the corresponding miRNAs were enriched in metastatic TN breast cancer and could be transferred into other cells to alter the expression of target genes.

**Table 2:** Functional studies of exosomal miRNAs related to breast cancer development

miRNAs	Target genes	Donor	Recipient	Functions	References
miR-141, miR-200a/b/c, miR-429		Cancer cells (4TO7)	Cancer cells (4T1E)	Metastatic potential	[43]
miR-9, miR-155	*PTEN, DUSP14*	Breast cancer cells (MDA-MB-231)	Breast cancer cells (MCF-7)	Metastatic potential	[44]
miR-105	*ZO-1*	Breast cancer cells (MCF-10A & MDA-MB-231)	Endothelial cells	Destroy tight junction and increase vascular permeability	[45]
miR-939	*VE-cadherin*	Breast cancer cells (MDA-MB-231)	Endothelial cells	Destroy the barrier function of endothelial monolayers	[46]
miR-181c	*PDPK1*	Breast cancer cells (MDA-MB-231, D3H2LN)	Endothelial cells	Destroy the blood-brain barrier Promote brain metastasis	[47]
miR-19a	*PTEN, CCL2*	Astrocyte	Breast cancer cells (MDA-MB-231, HCC1954)	Promote brain metastasis Increase proliferation and reduce apoptosis	[48]
miR-10b	*HOXD10, KLF4*	Breast cancer cells (MDA-MB-231)	Epithelial cells	Increase migration and invasion	[49]
miR-23b	*MARCKS*	Mesenchymal stem cells	Breast cancer cells (MDA-MB-231)	Induce dormancy in bone marrow	[51]
miR-127, miR-107, miR-222, miR-223	*CXCR12*	Mesenchymal stem cells	Breast cancer cells (MDA-MB-231, T47D)	Induce dormancy in bone marrow	[52]
miR-222, miR-223		Mesenchymal stem cells	Breast cancer cells (MDA-MB-231, T47D)	Induce dormancy in bone marrow	[53]
miR-223	Mef2c-β-catenin	Breast cancer cells (SKBR3)	IL-4 activated macrophages	Increase cell invasion	[54]
miR-21, miR-378e, miR-143		Fibroblast	Breast cancer cells (MDA-MB-231, T47D, BT549)	Increase EMT phenotype and stemness properties	[55]
miR-122	*PKM, CS*	Breast cancer cells (MDA-MB-231)	Fibroblast, neurons, microglia	Reprogrammed glucose metabolism	[57]
Let-7, miR-23b, miR-27a/b, miR-21, miR-320	PLAU*, AMOTL1*, NRP1*, ETS2*	Breast cancer cells (MCF7, MDA-MB-231)	Endothelial cells	Angiogenesis	[58]
miR-134	*STAT5B, Hsp90*	Breast cancer cells (Hs578Ts(i)8)	Breast cancer cells (Hs578T)	Decrease migration and invasion	[77]
miR-503	*CCND2, CCND3*	Breast cancer cells (MDA-MB-231)	Endothelial cells	Decrease cell proliferation and invasion	[78]

*Only the top differential expressed miRNAs were shown

During multiple steps of the metastatic process, cancer cells lose adhesion to the extracellular matrix and migrate into the circulation to reach the pre-metastatic niche. Exosomal miRNAs are implicated in these processes, which include cell migration, invasion, angiogenesis, bone marrow dormancy and reprogrammed metabolism. Exosomal miR-105 is reported to be released by cancer cells, which then destroys tight junctions and the integrity of natural barriers against metastasis^[45]^. MiR-105 is considered to be a potent regulator of migration, acting by targeting the tight junction protein ZO-1, leading to increased vascular permeability in distant organs. Similarly, the role of exosome-associated miR-939 in breast cancer has also been investigated^[46]^. This gene is a candidate to target VE-cadherin, a component of the adherens junctions involved in vessel permeability. The authors revealed that miR-939 was found to be highly expressed in TN subtypes catalogued in The Cancer Genome Atlas (TCGA) dataset. In the analysis of their cohort of 63 TN breast cancer patients, patients with high expression of miR-939 and nodal involvement showed a 6-fold higher risk of relapse compared to those without. Further, analysis of TN breast cancer cell lines showed that miR-939 directly targets VE-cadherin leading to an increase in permeability of a monolayer of human umbilical vascular endothelial cells.

MiR-181c is involved in migration of cancer cells through the blood–brain barrier (BBB)^[47]^. Tominaga *et al*.^[47]^ demonstrated that injection of brain metastatic cancer cell-derived exosomes into the tail vein of mice resulted in more brain metastasis compared with mice treated with human mammary tumor cell-derived exosomes.The exosome-derived miR-181c promotes destruction of the BBB through the abnormal localization of actin via downregulation of its target gene, 3-phosphoinositide-dependent protein kinase-1 (PDPK1). In analysis of breast cancer patients (*n* = 56), miR-181c in exosomes collected from brain metastasis patients (*n* = 10) was significantly higher compared with non-brain metastasis patients (*n* = 46), suggesting an important role of miR-181c in clinical utility for brain metastatic cancer. In addition, astrocyte-derived exosomal miR-19a has been reported to contribute to brain metastasis^[48]^. Zhang *et al*.^[48]^ showed that breast tumors after dissemination to the brain lost PTEN expression, which was regulated by miRNAs from astrocytes. Astrocyte-derived exosomal miR-19a reversibly downregulated PTEN expression in cancer cells, leading to increased secretion of cytokine chemokine (C-C motif) ligand 2 (CCL2) and recruitment of myeloid cells to enhance outgrowth of brain metastatic tumor cells. Their findings signify the dynamic and reciprocal cross-talk between tumor cells and the metastatic niche.

Another study by Singh *et al*.^[49]^ demonstrated that miR-10b was highly expressed in metastatic breast cancer MDA-MB-231 cells compared to non-metastatic breast cancer cells. Also, exosomal miR-10b secreted from MDA-MB-231 cells increased the invasive ability of nonmalignant immortalized human mammary epithelial cells. In particular, neutral sphingomyelinase 2 (nSMase2) or ceramide promoted secretion of exosomal miR-10b which then suppressed the protein level of its target genes such as Homeobox D10 (*HOXD10*) and Kruppel Like Factor 4 (*KLF4*), indicating functional significance.

### Exosomal miRNAs and dormancy in bone marrow

Breast cancer patients often experience recurrence decades after initial treatment and this seems to involve dormancy in the bone marrow^[50]^. Currently, there is growing research interest focusing on how different microenvironments affect bone marrow dormancy, and exosomes can play a crucial role in this. One study has revealed an association of miR-23b with bone marrow dormancy^[51]^. Ono *et al*.^[51]^ generated a bone marrow-metastatic human breast cancer cell line (BM2). Co-culture of BM2 and bone marrow mesenchymal stem cells (BM-MSCs) revealed suppression of proliferation, a decrease in stem cell-like properties, and inhibition of invasion in BM2 cells. MiR-23b derived from BM-MSCs can induce dormancy through the suppression of a target gene, myristoylated alanine-rich C kinase substrate (*MARCKS*), which encodes a protein that promotes cell cycling and motility. In a cohort of 10 patients, miR-23b expression was increased in metastatic bone lesions compared to matched primary breast tumors. In another study, bone marrow stromal cell-derived miR-127, miR-197, miR-222, and miR-223 led to cancer cell quiescence via down-regulation of CXCR12, a chemokine that interacts with CXR4 and CXR7 receptors^[52]^. Cancer cells entering quiescence or cell cycle arrest could acquire resistance to chemotherapy^[53]^. Bliss *et al*.^[53]^ revealed that breast cancer cells prime mesenchymal stem cells to release exosomes containing miR-222/223, which in turn promotes quiescence in a subset of breast cancer. In an immunodeficient mouse model of dormant breast cancer, therapy with antagomiR-222/223 sensitized breast cancer cells to carboplatin-based therapy and increased host survival.MiR-223 is also regarded as a specific miRNA for tumor-associated macrophages^[54]^. In a co-culture system, miR-223 released from IL-4-activated macrophages could be transferred to MCF-7 and MDA-MB-231 cells, leading to promotion of invasion of breast cancer cells via disruption of the Mef2c-β-catenin pathway^[54]^.

### Exosomal miRNAs modulate tumor microenvironment

Cancer-associated fibroblasts (CAFs) are vital constituents of the tumor microenvironment and play a major role in cancer initiation, angiogenesis, invasion, and metastasis of breast cancer^[54]^. Donnarumma *et al*.^[55]^ have demonstrated that breast cancer cells exposed to CAF exosomes containing miR-21, miR-378e, and miR-143, exhibited a significantly increased capacity to promote stemness properties, EMT phenotype, and anchorage-independent cell growth. Thus, the release of CAF exosomes may be responsible for the delivery of miRNAs that promote oncogenic signaling in breast cancer cells. In a large cohort of TCGA database breast cancer patients (*n* = 744), the patients with lower levels of miR-378 had longer overall survival, suggestive of a prognostic role for this gene.

### Exosomal miRNAs and glucose metabolism

Reprogrammed glucose metabolism is an emerging hallmark of cancer and several miRNAs have been implicated in metabolism and metabolic disorders^[56]^. Fong *et al*.^[57]^ showed that cancer cells suppress glucose uptake by non-tumor cells in the pre-metastatic niche, by secreting exosomal miR-122 that downregulates the target genes pyruvate kinase (*PKM*) and citrate synthase (*CS*). Both *in vitro* and *in vivo* studies have shown that exosomal miR-122 can reprogram the glucose metabolism of lung fibroblasts, brain astrocytes, and neurons that are abundantly present in the pre-metastatic sites of breast cancer.

### Exosomal miRNAs modulate angiogenesis

Some miRNAs are involved in modulation of angiogenesis. Hannafon *et al*.^[58]^ collected exosomes from MCF-7 and MDA-MB-231 breast cancer cells after treatment with docosahexaenoic acid (DHA). Several miRNAs (let-7a, miR-23b, miR-27a/b, miR-21, let-7, and miR-320b) were increased by DHA treatment in exosomes from breast cancer cell lines, but not in exosomes from normal breast cells. Exosomes derived from DHA-treated MCF-7 cells increased expression of the above miRNAs in recipient endothelial cells. Furthermore, the transfection of miR-23b and miR-320b into endothelial cells decreased expression of their pro-angiogenic target gene (*PLAU*, *AMOTL1*, *NRP1* and *ETS2*) and significantly inhibited tubular formation. These data suggest that the miRNAs transferred by exosomes mediate the anti-angiogenic action of DHA and support its potential use in cancer therapy. These findings demonstrate the capacity of exosomes derived from mesenchymal stem cells (MSCs) in the tumor microenvironment to exert antitumor effects by down-regulating vascular endothelial growth factor expression in recipient cancer cells.

## Exosomal micrornas related to drug response

### Resistance to endocrine therapy

The current treatment option for breast cancer varies according to tumor subtype or disease stage, which includes chemotherapy, endocrine therapy, molecular-targeted therapy, surgery, and radiotherapy. Approximately 70% of breast cancer tumors express ER and patients with ER-positive tumors are candidates for endocrine therapy. It is a major issue if cancer cells acquire resistance to endocrine therapy. There have been several studies analyzing miRNAs related to treatment resistance^[59]^, but there are only limited reports focusing on the exosomal miRNAs in resistance to endocrine therapy; we found only one article focused on this topic. Wei *et al*.^[60]^ suggested that exosomal miR-221/222 may be responsible for tamoxifen resistance in breast cancer. They generated tamoxifen-resistant MCF-7 (TamR) cells and compared them to tamoxifen-sensitive MCF-7 (wt) cells. They found that exosomes derived from MCF-7 (TamR) cells were able to enter into MCF-7 (wt) cells, enhancing tamoxifen resistance in recipient MCF-7 cells. The elevated miR-221/222 effectively reduced the expression of the target genes *P27* and *ERα*. These findings are supported by another study by Miller *et al*.^[61]^, revealing that tamoxifen-resistant breast cancer cells display up-regulation of miR-221/222 and significant reductions in *p27* levels. Taken together, these findings suggest that anti-miR-221/222 may be a potential therapeutic target to overcome tamoxifen resistance.

### Resistance to chemotherapy and molecular-targeted therapy

Anthracycline and taxane-containing agents are most commonly used as adjuvant chemotherapy for early breast cancer^[62]^, but their efficacy is often limited by the emergence of chemoresistance^[63]^. The acquisition of chemoresistance requires multiple regulatory changes of the tumor microenvironment, some of which are caused by exosomes. To improve the clinical outcome of cancer patients, accurate biomarkers for early prediction of response or resistance to chemotherapy are needed.

There have been few studies concerning the association between exosomal miRNAs and treatment efficacy in the clinical setting. We found one article by Stevic *et al*.^[37]^ on this topic. They analyzed several exosomal miRNAs which were determined based on analysis with a microRNA array card using the patients’ plasma samples before neoadjuvant therapy (GeparSixto trial). The expression of miR-155 and miR-301 in exosomes of HER2-positive and TN breast cancer patients most significantly predicted pathological complete response (pCR) which is considered as a surrogate marker for prognosis, in uni- and multivarite models. MiR-301 regulates the phosphatase and tensin homolog (PTEN)/Akt and nuclear factor-kappa B (NF-κB) signaling pathways^[64,65]^ and also binds to the *ESR 1* gene, leading to estrogen-independent growth of breast cancer^[66]^.

**Figure 1:**
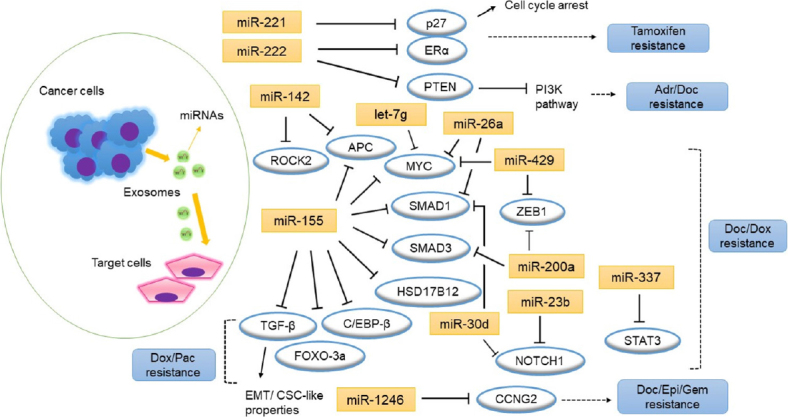
MicroRNAs and the putative target genes associated with drug resistance in breast cancer cells. Arrows indicate activation and bar indicate inhibition. Adr: adriamycin; CSC: cancer stem cell; Doc: docetaxel; Dox: doxorubicin; EMT: epithelial-to-mesenchymal transition; Epi: epirubicin; Gem: gemcitabine; Pac: paclitaxel

In contrast, we identified several functional studies using cell lines to investigate the association of exosomal miRNAs with drug response [Table 3 and Figure 1]. Basically, it is emphasized that drug-resistant breast cancer cells can deliver exosomal miRNAs to sensitive cells to spread resistance. In some studies, individual exosomal miRNAs were examined. Li *et al*.^[67]^ focused on the exosomal miR-1246, which has been reported to function as a protooncogene in lung and other cancers^[67]^. In their study, miR-1246 was highly expressed in metastatic breast cancer MDA-MB-231 cells compared to non-metastatic breast cancer cells or non-malignant breast cells^[67]^. Further, the transferred miR-1246 promoted invasion in non-malignant human mammary epithelial (HMLE) cells in part by targeting Cyclin G2 (CCNG2). The HMLE cells, after treatment with exosomes from MDA-MB-231 cells transfected with miR-1246, gained resistance to docetaxel, epirubicin, and gemcitabine.

**Table 3:** Exosomal miRNAs related to response to drug in breast cancer

miRNAs	Target genes	Sample source		Drug	Functions	References
		Cell lines	Patients			
miR-221, miR-222	*p27, ERα*	MCF-7		Tamoxifen	MiR-221/222 enhance tamoxifen resistance in recipient cells.	[60]
miR-155, miR-301			Plasma	Paclitaxel, doxorubicin, carboplatin	Predict pathological complete response (pCR)	[37]
miR-1246	*CCNG2*	MDA-MB-231		Docetaxel, epirubisin, gemcitabine	Cells transfected with miR-1246 induce drug resistance	[67]
miR-222	*PTEN*	MCF-7		Adriamycin, docetaxel	High expression levels of miRNAs correlate to drug-resistance cells	[68,69]
miR-100, miR-30a, miR-17		MCF-7		Adriamycin, docetaxel	High expression levels of miRNAs correlate to drug-resistant cells	[68]
miR-1246, miR1268a, miR-149-3a, miR-423-5a, miR-4298, miR-4438, miR-4644, miR-671-5p, miR-7107-5p, miR7847		MDA-MB-231		Docetaxel	Ten miRNAs were up-regulated in docetaxel-resistant cells	[70]
miR-138-5p, miR-139-5p, miR-197-3p, miR-210-3p, miR-3178, miR423-5p, miR-4258, miR-4443, miR-574-3p, miR-6780b-3p, miR-744-5p		MDA-MB-231		Epirubicin	Eleven miRNAs were up-regulated in epirubicin-resistant cells	[70]
miR-138-5p, miR-140-3p, miR-210-3p, miR-3613-5p		MDA-MB-231		Vinorelbine	Four miRNAs were up-regulated in vinorelbine-resistant cells	[70]
miR-23a-3p, miR-27a-3p, miR-30a-5p, miR-320a, miR-455-3p*		MCF7		Adriamycin	Adriamycin-resistant cells correlate to pathway of “transcriptional misregulation in cancer“	[71]
miR-142-3p, miR-155-5p	*APC*	MCF10A/HCC1806-EVs		Docetaxel, doxorubicin	Correlate to drug-resistant cells	[72]
miR-155-5p	*HSD17B12*	MCF10A/HCC1806-EVs		Docetaxel, doxorubicin		[72]
let-7g-5p, miR-155-5p, miR-26a-5p, miR-429	*MYC*	MCF10A/HCC1806-EVs		Docetaxel, doxorubicin		[72]
miR-23b-3p, miR-30d-5p	*NOTCH1*	MCF10A/HCC1806-EVs		Docetaxel, doxorubicin		[72]
miR-142-3p	*ROCK2*	MCF10A/HCC1806-EVs		Docetaxel, doxorubicin		[72]
miR-155-5p, miR-26a-5p, miR-30d-5p	*SMAD1*	MCF10A/HCC1806-EVs		Docetaxel, doxorubicin		[72]
miR-155-5p, miR-200a-3p	*SMAD3*	MCF10A/HCC1806-EVs		Docetaxel, doxorubicin		[72]
miR-337-3p	*STAT3*	MCF10A/HCC1806-EVs		Docetaxel, doxorubicin		[72]
miR-1236-3p, miR-200a-3p, miR-23b-3p, miR-429	*ZEB1*	MCF10A/HCC1806-EVs		Docetaxel, doxorubicin		[72]
miR-200a-3p, miR-429	*ZEPM2*	MCF10A/HCC1806-EVs		Docetaxel, doxorubicin		[72]
miR-155	*TGF-β, FOXO-3a, C/EBP-β*	MCF-7, MDA-MB-231		Doxorubicin, paclitaxel	MiR-155 transfected cells displayed EMT change and resistance to drug	[73]
miR-134	_	Hs578Ts(i)8	_	Anti-Hsp90 drug	The cells transfected with miR-134 increased sensitivity to anti-Hsp90 drug	[77]

* Only the top differential expressed miRNAs were shown

MiR-222, miR-100, and miR-30a are reported to contribute to drug resistance of MCF-7 cell lines^[68,69]^. Chen *et al*.^[68]^ generated two drug resistance models; MCF-7/adriamycin (Adr) and MCF-7/docetaxel (Doc), to investigate the mechanisms of chemotherapy failure. They demonstrated that following transfer of exosomes derived from MCF-7/Adr and MCF-7/Doc to recipient MCF-7/sensitive cells, the levels of miR-100, miR-222, miR-30a, and miR-17 were all significantly increased in the recipient cells. Further, MCF-7/Adr and MCF-7/Doc displayed a marked reduction of PTEN, which is a target of miR-222, compared to MCF-7/sensitive cells. Zhong *et al*.^[70]^ established three resistant cell lines by exposing the parental MDA-MB-231 cell line to docetaxel (Doc), epirubicin (Epi), and vinorelbine (Vin), respectively^[70]^. They found some miRNAs were dysregulated in resistant cell lines relative to sensitive MDA-MB-231 cells; there were 10 consistently up-regulated miRNAs in MDA-MB-231/Doc resistant cells and their exosomes, 11 in MDA-MB-231/Epi resistant cells, and four in MDA-MB-231/Vin resistant cells (gene lists are presented in Table 3). Further, 12 of the above 22 miRNAs were significantly up-regulated after neoadjuvant chemotherapy but this change was observed in exosomes and not in tissues. Pathway analysis revealed that the predicted target genes of the corresponding miRNAs were enriched in 17 pathways, which included p53, Wnt, mitogen-activated protein kinase (MAPK), and ErbB2 signaling pathway.

Similarly, Chen *et al*.^[71]^ attempted to comprehensively evaluate miRNA expression profiles relevant to chemoresistance using bioinformatic studies. They found that a total of 309 miRNAs were increased and 66 miRNAs were decreased significantly in adriamycin-resistant breast cancer cells compared with parental ones. The top up-regulated miRNAs were miR-23a-3p, miR-27a-3p, miR-30a-5p, and miR-320a and the top down-regulated miRNA was miR-455-3p. KEGG analysis based on the most abundant 13 miRNAs related to adriamycin-resistant cells provided further information that “transcriptional misregulation in cancer” was the most prominent pathway.

Ozawa *et al*.^[72]^ focused on the mechanisms of action of exosomes derived from TN breast cancer cells with the most aggressive phenotype. When non-tumorigenic breast cells MCF-10A were treated with exosomes derived from HCC1806 TN breast cancer cells, they found a significant increase in cell proliferation and resistance to the therapeutic agents tested (doxorubicin and docetaxel). Gene and miRNA expression profiling revealed 138 genes and 70 miRNAs were significantly differentially expressed among the corresponding MCF-10A and the untreated MCF-10A cells, affecting mostly the phosphoinositide 3-kinase (PI3K)/AKT, MAPK, and hypoxia inducible factor (HIF)1A pathways. Ten of these miRNAs control some genes [Table 3], and miR-155 was the one that regulates the greatest number of targets [Figure 1]; which were adenomatosis polyposis coli (*APC*), hydroxysteroid 17-β dehydrogenase 12 (*HSD17B12*), *MYC*, *SMAD1* and *SMAD3*. The relevance of the corresponding genes was also shown by another study^[73]^. They found miR-155 induction in exosomes isolated from cancer stem cells and resistant cells^[73]^. When miR-155 exosomes were transfected into recipient sensitive cells, they underwent EMT and gained intermediate resistance to doxorubicin and paclitaxel therapy. It is well-known that miR-155 mediates the loss of CCAAT enhancer-binding protein (C/EBP)-β^[74,75]^, which in turn, causes loss of transforming growth factor (TGF)-β and leads to EMT in breast cancer cells^[76]^. They also observed repression of TGF-β, C/EBP-β and forkhead box O (FOXO)3a in cells transfected with miR-155^[73]^.

O’Brien *et al*.^[77]^ reported the association of miR-134 with drug sensitivity. They identified miR-134 as the most substantially down-regulated miRNA in the aggressive TN breast cancer cells, Hs578Ts(i)8 and their exosomes compared to their parental (Hs578T) counterparts. In analysis of clinical samples, miR-134 was found to be significantly down-regulated in breast cancer when compared to its levels in healthy breast tissue. Further, the transfection of miR-134-enriched exosomes into Hs578Ts(i)8 cells reduced the levels of its target genes, signal transducer and activator of transcription 5B (*STAT5B*) and heat shock protein 90 (*Hsp90*), leading to a reduction in migratory and invasion ability. The corresponding cells showed increased sensitivity to an anti-Hsp90 drug. Their functional studies support the potential use of miR-134 as a therapeutic agent in TN breast cancer.

As described in this section, there is accumulating evidence supporting an association between exosomal miRNA and resistance to anthracycline or taxane-containing agents in breast cancer cells. There are few reports of exosomal miRNAs on the effects of molecular-targeted therapy. It remains a challenge for future research to explore its association with other drugs, such as eribulin or anti-HER2 therapy.

## Liminations of research into exosomal MicroRNAs

Exosomal miRNAs can be attractive future diagnostic markers and therapeutic targets but there remain some unsolved problems which must be overcome before they can be applied in clinical practice. First, exosome research is based on the findings that a higher secretion of exosomes may be a general feature of cancer patients compared to healthy individuals and miRNAs encapsulated in exosomes reflects originating tumor biology. Nevertheless, some studies have suggested that miRNAs derived from exosomes could not parallel those in primary tumors^[42]^. For example, Bovy *et al*.^[78]^ examined the expression levels of exosomal miR-503 in patients after neoadjuvant chemotherapy. Interestingly, these levels increased after therapy, whereas miR-503 in primary tumors subjected to this treatment did not change. The authors speculated that the increased miR-503 levels in the circulation after chemotherapy did not originate from the tumor, but from the endothelial cells. Exosomes can be secreted by various types of cells. It will be ideal if cancer-specific exosomes or exosomal miRNAs can be more accurately isolated to investigate how donor tumor cells select or sort the packaging of miRNAs into exosomes to regulate their progression. Several efforts to solve this issue have been undertaken, involving surface protein markers on exosomes, such as CD24 or EpCAM^[79]^, but no standard markers to differentiate cancer-specific exosomes from those of other origin have been identified.

Second, there is no reliable endogenous gene for normalization of exosomal miRNAs. According to several reports, miR-16-5p, miR-423-3p and miR-191-5p are often recommended as reference miRNAs, since they are constantly expressed through the data^[27,80]^. Meanwhile snRNU6 is not detectable in most blood samples. However, these reference genes could not always fit exclusively to individual samples with different populations and characteristics. We therefore need to assess them within each dataset. As another method, most suitable miRNAs can be tested by software such as the Genorm Algorithm^[37]^. In addition to endogenous normalization, exogenous normalization (e.g., cel-miR-39) to remove the technical and interindividual variability are required. Anyway, currently there are no unified and standard reference genes in exosome research.

Finally, there are difficulties in interpretation and statistical analysis of obtained data. Recent studies tend to examine comprehensive expression of exosomal miRNAs such as microarray or next-generation sequencing (NGS), which consequently offer us enormous data regarding candidate miRNAs. It is difficult to determine whether all the differentially-expressed miRNAs have a meaningful role in the process of cancer development. In addition, one miRNA has binding affinity to numerous mRNAs and is involved in the downregulation of many factors that participate in different cancer-relevant signal transduction pathways. Some miRNAs, such as miR-155 and miR-335, have the potential to exert both tumor-suppressive and oncogenic effects^[81,82]^, which confuses the understanding of their biological mechanisms. To explore the biological functions of identified miRNAs, bioinformatic analysis using programs such as TargetScan, miRbase, miRWalk, DIANA-microT and TarBase are needed.

## Conclusion

Investigation of the involvement of exosomal miRNAs in breast cancer could provide promising diagnostic and prognostic biomarkers as well as increasing understanding of the biological mechanisms of cancer development. There is accumulating evidence regarding the role of exosomal miRNAs, especially in functional studies, whereas evidence of their role in the clinical setting remains inadequate. We found few overlapping exosomal miRNAs between the studies, so a large clinical cohort may be required to obtain reproducible data.

With regard to experimental studies, it is of interest that not only cancer cells but surrounding cells such as endothelial cells, pericytes, fibroblasts, and immune cells, secrete exosomes to modulate tumor-favorable or -unfavorable conditions. Considering the potential of exosomes as cancer biomarkers, it is becoming necessary to understand the many findings related to the tumor microenvironment. Although individual miRNAs have been abundantly explored as we describe above, the entry of exosomal miRNAs into clinical practice is hampered since not all the identified miRNAs have been fully checked using clinical samples. To expand the research field into clinical studies could help identify those patients who are most likely to experience recurrence or who are most likely to acquire resistance or respond to therapy. Breast cancer tumors are characterized by extensive heterogeneity (temporal or spatial) and comprise several subtypes with different molecular profiles^[83]^, which makes it difficult to understand the comprehensive features of disease conditions and identify unique targets. A panel of specific miRNAs as real-time biomarkers, in combination with other conventional clinical biomarkers, might improve the diagnostic and predictive power. We believe that this review will provide the opportunity for a more critical evaluation of the clinical value of exosomal miRNAs.

## Declarations

### Copyright

© The Author(s) 2019.
